# Joint Prior Learning for Visual Sensor Network Noisy Image Super-Resolution

**DOI:** 10.3390/s16030288

**Published:** 2016-02-26

**Authors:** Bo Yue, Shuang Wang, Xuefeng Liang, Licheng Jiao, Caijin Xu

**Affiliations:** 1 Key Laboratory of Intelligent Perception and Image Understanding of Ministry of Education, International Research Center for Intelligent Perception and Computation, Joint International Research Laboratory of Intelligent Perception and Computation, Xidian University, Xi’an 710071, China; yuebo312@live.com (B.Y.); jlcxidian@163.com (L.J.); xcj0918xd.@163.com (C.X.); 2 Department of Intelligence Science and Technology, Graduate School of Informatics, Kyoto University, Kyoto 606-8501, Japan

**Keywords:** visual sensor network, image super-resolution, image denoising, prior learning, EM algorithm

## Abstract

The visual sensor network (VSN), a new type of wireless sensor network composed of low-cost wireless camera nodes, is being applied for numerous complex visual analyses in wild environments, such as visual surveillance, object recognition, *etc*. However, the captured images/videos are often low resolution with noise. Such visual data cannot be directly delivered to the advanced visual analysis. In this paper, we propose a joint-prior image super-resolution (JPISR) method using expectation maximization (EM) algorithm to improve VSN image quality. Unlike conventional methods that only focus on upscaling images, JPISR alternatively solves upscaling mapping and denoising in the *E-step* and *M-step*. To meet the requirement of the *M-step*, we introduce a novel non-local group-sparsity image filtering method to learn the explicit prior and induce the geometric duality between images to learn the implicit prior. The *EM* algorithm inherently combines the explicit prior and implicit prior by joint learning. Moreover, JPISR does not rely on large external datasets for training, which is much more practical in a VSN. Extensive experiments show that JPISR outperforms five state-of-the-art methods in terms of both PSNR, SSIM and visual perception.

## 1. Introduction

A large or small visual sensor network (VSN) depending on spatially-distributed smart cameras is for sensing, communicating and fusing images of a scene from varied viewpoints. It has been applied to a variety of public applications, including security and area surveillance, tracking, environmental monitoring, *etc.* A VSN is generally equipped with low-cost camera nodes, which are simply stuck on walls or poles in a wild environment, such as dark lighting and dirt on the lens. Due to the working condition and limitations of the low-cost imaging acquisition device, most of the obtained frames are low resolution (LR) images with certain noise. Even a small amount of noise, which is inevitable in low-light conditions, reduces the visibility of details that could contain vital information.

In a VSN, one desires to obtain high resolution (HR) images with the least noise. However, constructing the HR image x from the observed LR image y with noise is a typically ill-posed. Mathematically, it can be modeled as:

(1) y=AHx+n

AHx+n is the mapping function of the image sensor from a real scene to the LR image. More specifically, A denotes the downsampling process; H is a blurring operation; and n represents the additive white Gaussian noise with variance $\delta^{2}$. Solving this problem is indeed a non-trivial challenge due to the fewer rows than columns in the matrix AH.

To address this ill-posed issue, the prior information about the desired HR image may convert this problem into being well-posed. In recent years, this information has generally been divided into two categories: the explicit prior and the implicit prior. The first model reflects our basic understanding or assumption of the distribution or energy function of the target reconstructed image. It formulates the problem within the Bayesian framework by maximizing the probability of the HR image from the LR image. It can be generally modeled as:

(2) $$x=\arg\min_{x}{∥y-\mathrm{AH}x∥}_{2}^{2}+\lambda R\left(x\right)$$

where ${∥y-\mathrm{AH}x∥}_{2}^{2}$ is the likelihood function describing the probability relation between the LR image y and the original HR image x, $R\left(x\right)$ is the prior knowledge of the HR image showing how likely a reconstructed image x is. Therefore, the optimization strategy [1,2,3] and the selection of prior information [4,5,6,7,8,9,10] have been the core issues of explicit prior learning.

As opposed to explicit prior learning, implicit prior learning is somehow non-selective. The HR image prior can be implicitly defined, rather than explicitly given by the specific statistical distribution. It is intimately related to the learning-based super-resolution, which aims to learn the co-occurrence of local structures between the LR and HR images from the external/internal training database. Recently, two important foundations have been proposed, namely the coupled overcomplete dictionary learning [11,12,13,14] and the deep networks [15,16], for image super-resolution. They either learn an LR-HR overcomplete dictionary pair or network parameters from training datasets that contain a million co-occurrences of LR-HR image patches. Additionally, then, the dictionary pair or network parameter is the implicit prior to estimate the nonlinear mapping between the LR and HR images. The general model is:

(3) $$\begin{array}{c} \begin{array}{cc} x & =F\left(y;w\right)\\ \text{s.t.}\;w=\arg\min_{w} & {∥x_{tr}-F\left(y_{tr};w\right)∥}_{p}^{2}+\lambda R\left(w\right) \end{array} \end{array}$$

where $x_{tr},\;y_{tr}$ are the training data, F represents the mapping function, w is the dictionary pair or network parameters and $R\left(w\right)$ is the penalty on the parameters.

One can see that neither an explicit prior nor an implicit prior can completely super-resolve the noisy LR images in a VSN; because both of them concentrate on the upscaling mapping from the LR image to the HR image, but consider the denoising less. Moreover, they require large datasets for model training. This is almost impossible and unnecessary in a VSN. In this paper, we propose a joint-prior image super-resolution (JPISR) method using the expectation maximization (EM) algorithm for image quality improvement in a VSN. This EM algorithm alternatively solves upscaling mapping and denoising in the *E-step* and *M-step*. Specifically, we introduce a maximum a posteriori (MAP)-based HR image estimation method for the *M-step*, where the explicit prior serves as the likelihood estimation, and the implicit prior is regarded as the Bayesian prior estimation; in which we introduce a novel adaptive non-local group-sparsity image filtering method for likelihood estimation to adequately mine the explicit prior. Meanwhile, we induce the geometric duality [17,18] between the LR and HR images/patches into the implicit prior learning about every individual clean image patch, which can further enhance the super-resolution performance. Then, one can predict the mean and covariance of the target image patch from its respective LR image patch by a *Gaussian process* [19,20]. JPISR inherently integrates the above two priors’ learning into one framework. Thanks to the non-local group-sparsity and the geometric duality, this joint learning does not require external training data. Finally, we obtain the posterior of the HR image patch by the *Bayesian minimum mean-square error (BMMSE) estimator*.

The contributions of this paper can be summarized as follows:

- We propose a JPISR method based on the expectation maximization (EM) algorithm for image quality improvement in a visual sensor network, which can effectively reconstruct finer details and simultaneously suppress noises for smart sensing.
- In the *M-step* procedure, the novel adaptive non-local group-sparsity explicit prior serves as the likelihood estimation, and the geometric duality implicit prior is regard as the Bayesian prior estimation. They are effectively integrated into one framework by maximum a posteriori (MAP). Since there is no need for the external training data, this joint prior learning is very suitable for a VSN.
- When a pattern with a high frequency signal is simple, but rarely repeated in the image, we introduce a rotation invariance into non-local self-exemplars to increase the number of repeated image patches for explicit prior learning.

The rest of this paper is organized as follows. We briefly review the related visual sensor network and the state-of-the-art image super-resolution techniques in Section 2. Then, in Section 3, we explain the proposed EM algorithm for joint-prior learning image super-resolution in detail. Section 4 introduces the adaptive non-local group-sparsity image filtering method and the implicit prior learning by the *Gaussian process*. In Section 5, experimental results and comparisons with state-of-the-art methods are provided to show the effectiveness of the proposed method. Finally, we conclude the paper in Section 6.

## 2. Related Works

The visual sensor network has recently emerged as a new type of sensor-based intelligent system, which processes the captured image/video data locally and collaborates with other cameras over a network. Taking into account the high production cost of cameras, the VSN is often equipped with low-cost cameras. For the applications in wild environments, the LR image with noise seriously restricts the efficiency of the VSN, due to the targets being covered by a few pixels in size [6,21,22]. Thus, image super-resolution methods are desired to enhance the image quality.

As previously mentioned, the explicit prior and the implicit prior could make the image super-resolution problem well-posed. The explicit prior learning can be interpreted from the *Bayesian* perspective. Babacan *et al.* [1] proposed a variational Bayesian method to estimate the distributions of all unknowns. Zhao *et al.* [3] proposed a new fast super-resolution approach, which is successfully embedded into the alternating direction method of multipliers (ADMM) framework. Sun *et al.* [7] exploited the gradient profile priors for local image structures. Dong *et al.* [8] took advantage of the non-local similarity, sparse representation and autoregressive (AR) models in an image. Zhang *et al.* [9] modeled a natural image prior by a high-order Markov random field (MRF). Yang *et al.* [10] proposed a method that exploits self-similarities and group structural information of image patches. Li *et al.* [23] combined sparse representation and non-local similarity for image SR. Sajjad *et al.* [24] used an over-redundant dictionary based on effective image representations for image SR. The main differences among these methods are the optimization strategies and selections of prior information.

On the other hand, the implicit prior is learned from the mapping relationship from the LR image patch space to the HR image patch space. Considering that the image patches of different resolutions are of different linear spaces and each image patch can be represented by a vector, the relation between the LR image patch and HR image patch can be regarded as the relation between two vector spaces associated with the two image patches’ styles. Under this assumption, a series of learning (mapping)-based approaches has been proposed to model the relationship between the LR image patch and the HR image patch. As the seminal work, the Markov random field was proposed by Freeman *et al.* [25] to model the relationship between the HR image patch and the LR one and between the HR image patch and its neighboring patches. Then, the HR image is inferred by the likelihood maximum. The most well-known method is coupled dictionary learning based on sparse representation, which was proposed by Yang *et al.* [11,12]. As its core, the assumption is that the sparse representation of the HR image patch is the same as that of the LR one. Thus, the HR dictionary and the LR dictionary are united into a single dictionary, which is trained by classical dictionary learning methods. Considering that it is impossible to learn a universal model that creates such as a complex mapping relationship, Zhang *et al.* [14] proposed multiple linear mapping functions for super-resolution reconstruction. Recently, deep learning-based methods [15,16] were introduced to learn the relationship and show distinct advantage over existing state-of-the-art methods.

## 3. EM Scheme of Image Super-Resolution in a VSN

### 3.1. Problem Formulation

The low-cost VSN usually produces LR images/videos with noise. Image super-resolution aims to transform the LR image to the HR image, which is modeled in Equation (1). However, most super-resolution methods assume that input LR images have no noise. This is far from the reality in a VSN. Moreover, directly super-resolving the noisy LR image has little practicability. To address this issue, we introduce a missing datum, the so-called hidden variable (image) z, by dividing the image super-resolution degradation procedure into two problems:

(4) $$\left\{\begin{array}{c} z=x+\alpha n_{1}\\ y=Sz+n_{2} \end{array}\right.$$

where we lump the downsampling operator and blurring operator into a single measurement matrix S=AH, $n_{1}∼N\left(0,I\right)$ and $n_{2}∼N\left(0,\delta^{2}I-\alpha^{2}SS^{T}\right)$ are independent Gaussian noises, such that $n=\alpha Sn_{1}+n_{2}∼N\left(0,\delta^{2}I\right)$, and *α* (α<δ) is a positive parameter controlling the distribution of noise.

Clearly, if z is given, we could obtain the HR image x by solving the equation $z=x+\alpha n_{1}$, which is a pure denoising problem, where $\alpha n_{1}$ represents zero-mean noise with covariance $\alpha^{2}I$. This key observation lets us be able to treat z as a missing datum and alternately estimate x and z in the *E-step* and *M-step* for simultaneous image upscaling and denoising.

### 3.2. EM Scheme

#### 3.2.1. *E-Step*: The Likelihood of the Super-Resolution Model Approach Procedure

The *E-step* finds the conditional expectation value of the complete-data log-likelihood $\log p\left(y,z|x\right)$ with respect to the unknown z, the observed data y and the current parameter (the estimated HR image) for ${\hat{x}}^{t}$. The so-called *Q*-function is defined as follows:

(5) $$\begin{array}{c} Q\left(x,{\hat{x}}^{t}\right)=E\left[\log p\left(y,z|x\right)|y,{\hat{x}}^{t}\right] \end{array}$$

Equation (5) can be further reformulated as follows:

(6) $$\begin{array}{c} Q\left(x,{\hat{x}}^{t}\right)=-\frac{1}{2\alpha^{2}}{∥x-{\hat{z}}^{t}∥}_{2}^{2}+k \end{array}$$

where ${\hat{z}}^{t}$ is the *t*-th hidden image estimate z and *k* is a constant. The proof of Equation (6) is given in [26]. The *t*-th hidden image estimate ${\hat{z}}^{t}$ can be derived as follows.

Let ${\hat{x}}^{t}$ be the *t*-th HR image estimation. The estimation of the *t*-th hidden image estimate ${\hat{z}}^{t}$ is:

(7) $$\begin{array}{c} {\hat{z}}^{t}={\hat{x}}^{t}+\frac{\alpha^{2}}{\delta^{2}}S^{T}\left(y-S{\hat{x}}^{t}\right) \end{array}$$

#### 3.2.2. *M-Step*: Image Denoising Procedure

The *M-step* is to maximize the expectation (*Q*-function) in the *E-step* by updating the estimated HR image x according to:

(8) $$\begin{array}{c} {\hat{x}}^{t+1}=\arg\max_{x}\left\{Q\left(x,{\hat{x}}^{t}\right)-q\left(x\right)\right\} \end{array}$$

where q(x) is a penalty function to x. When the log prior of x is used as the regularization, the optimization of Equation (8) becomes the MAP estimation,

(9) $$\begin{array}{c} {\hat{x}}^{t+1}=\arg\min_{x}\left\{∥x-{\hat{z}}^{t}{∥}_{2}^{2}+\gamma \log\mathrm{pr}\left(x\right)\right\} \end{array}$$

where pr(x) is the prior of x, and *γ* is the trade-off parameter. Thus, the *M-step* is indeed an image denoising procedure, which combines the reconstruction constraint and prior knowledge to further improve the quality of the estimated HR image.

Thus, the entire EM algorithm for solving noisy image super-resolution can be summarized as:

- Likelihood of the super-resolution model approach *(E-step)*:
  $$\begin{array}{c} {\hat{z}}^{t}={\hat{x}}^{t}+\frac{\alpha^{2}}{\delta^{2}}S^{T}\left(y-S{\hat{x}}^{t}\right) \end{array}$$
- HR image estimation from a hidden image *(M-step)*:
  $$\begin{array}{c} {\hat{x}}^{t+1}=\arg\min_{x}\left\{∥x-{\hat{z}}^{t}{∥}_{2}^{2}+\gamma \log\mathrm{pr}\left(x\right)\right\} \end{array}$$

One can see the *E-step* can be easily achieved because only a simple linear transformation combination is applied. It can also be considered as a likelihood approach or a gradient descent method. Thus, the major difficulty in our image super-resolution method is the *M-step*. That is how to estimate the HR image x from the hidden image z.

## 4. HR Image Estimation via Maximum A Posterior

As previously mentioned, *M-step* is an image denoising procedure. We then borrow the basic idea of the adaptive non-local group-sparsity methods [27,28], which perform effectively in image denoising. In our work, the non-local group-sparsity explicit prior serves as the likelihood estimation. It formulates the *M-step* as an optimal filter design problem and determines the spectral coefficients of the filter by considering a local Bayesian prior. However, this local Bayesian prior requires the statistical distribution of the image patch from a similar targeted database, which is not practical in a VSN. To alleviate this problem, we consider the geometric duality existing between the LR and HR image patches as the implicit prior and jointly learn it to predict the mean and covariance of the target image patch from its respective LR image patch, rather than external databases using the *Gaussian process* [19]. Thus, the explicit prior serving as the likelihood estimation and the implicit prior regarded as the prior estimation in *M-step* can be jointly learned by the MAP procedure.

### 4.1. Non-Local Group-Sparsity Explicit Prior Learning

HR image ${\hat{x}}^{t+1}$ estimation based on non-local group sparsity can be treated as a linear image patch denoising filter design procedure, which is described as below.

Given a noisy image patch $q\in R^{d}$ from the hidden image ${\hat{z}}^{t}$, estimate a linear transform operator (a filter) $A\in R^{d\times d}$ to make the estimation p-Aq have the *minimum mean squared error (MMSE)*:

(10) $$A=\underset{A}{\arg\min}E\left[{∥Aq-p∥}_{2}^{2}\right]$$

where p is the ground truth image patch. In general, we assume that A is symmetric and square. Thus, we can gain a better understanding of this linear transform by performing the *singular value decomposition* $A=U\Lambda U^{T}$, where the dictionary $U\in R^{d\times d}$ is an orthonormal matrix satisfying $U^{T}U=I$, and $\Lambda =\mathrm{diag}\left\{\lambda_{1},\ldots ,\lambda_{d}\right\}\in R^{d\times d}$ is the diagonal matrix containing the spectral coefficients. Therefore, the optimization problem Equation (10) is rewritten by:

(11) $$\left(U,\Lambda \right)=\underset{U,\Lambda }{\arg\min}E\left[{∥U\Lambda U^{T}q-p∥}_{2}^{2}\right]$$

If the dictionary U is known, the MAP estimator of Λ has a closed-form solution, especially when the dictionary U is square and unitary. $U^{T}q$ is an operation that transforms the image patch from the space domain to the frequency domain. The symbol Λ is an element-wise shrinkage operator of the transform spectrum, where the true signal component is reserved and the noise is suppressed by the element-wise operation. Thus, the true information can be successfully separated from the noise by shrinkage. The denoised image patch is then obtained by multiplication by U, which transforms from the frequency domain back to the space domain. Repeat this process until all of the image patches have been denoised.

Thus, it is natural to determine the basis matrix U and to compute the MAP estimator of Λ. However, constructing dictionary U of image patch q has two issues: what input should we use, and how do we train the dictionary? We give the solution below.

Similar to the idea of the dictionary learning strategy in [29], the input training samples are obtained directly from the similar patches ${\left\{p_{j}\right\}}_{j=1}^{k}$, and this is modeled in the unit of the group. In other words, we search the similar patches within the noisy image. The similar patch is selected if the Euclidean distance between the patch q and the patch $p_{j}$ is less than a threshold value. Each group ${\left\{p_{j}\right\}}_{j=1}^{k}$ is represented by the form of a matrix, denoted by P, which is composed of patches similar to the patch q.

With this first issue solved, we concentrate on the dictionary U learning from group P. Luo *et al.* [28] pointed out that a good dictionary U should satisfy the following two properties: first, the projected vector ${\left\{U^{T}p_{j}\right\}}_{j=1}^{k}$ should be similar in both magnitude and location, which is based on the observation that similar patches have a similar decomposition [27]; second, each projected vector $U^{T}p_{j}$ should be sparse. The more non-Gaussian image patches we have, the easier it is to distinguish from Gaussian noise because the noise is not sparse. Hence, this idea is more effective for denoising.

In order to satisfy the above-mentioned two criteria, we propose the group sparsity that is represented by a joint sparsity pattern; in which we introduce a so-called $\;\;\;\ell_{1,2}$ mixed norm matrix ${∥A∥}_{1,2}={\left(\sum_{i}{∥a_{i}∥}_{1}^{2}\right)}^{1/2}$ where $a_{i}$ is the *i*-th column of matrix A. Thus, the $\;\;\;\ell_{1,2}$ norm of the matrix $U^{T}P$ is minimized as follows:

(12) $$\begin{array}{c} \underset{U}{\mathrm{minmize}}\;\;\;\;\;\;\;\;\;{∥U^{T}P∥}_{1,2}\;s.t.\;U^{T}U=I \end{array}$$

where the objective function is to minimize the joint sparsity error, and the constraint is to impose the orthogonality of U. This problem seems to be complex, but actually is identical to *principal component analysis (PCA)* [28,29]. We then compute:

(13) $$\left[U,S\right]=\mathrm{eig}\left(PP^{T}\right)$$

where U is the eigenvector matrix and S denotes the corresponding eigenvalue matrix.

When the dictionary of U has been learned, we will compute the optimal Λ by the *Bayesian minimum mean-square error (BMMSE)*. Most image denoising techniques learn “universal” image priors from a variety of scenes to guide the denoising for all kinds of images. Then, the Λ is obtained by a simple hard thresholding whose value is empirically gained. Obviously, such Λ is less accurate. Luo [28] considered an image patch prior f(p) learned from the similar targeted database, which makes Λ much more specific and accurate. However, having a targeted database is not practical in a VSN. We then consider that the geometric duality exists between the LR and HR image patches as the implicit prior and jointly learn it to predict the mean and covariance of the target image patch from its respective LR image patch, rather than the external database using the *Gaussian process* [19].

To have f(p), we assume that the mean *μ* and covariance Σ of f(p) are known. Then, the optimal Λ is derived by the following lemma:

Let $f\left(q|p\right)=N\left(p,\alpha^{2}I\right)$, and let $f\left(p\right)=N\left(\mu ,\Sigma \right)$ for any vector *μ* and matrix Σ; then, the optimal Λ that minimizes Equation (11) is:

(14) $$\Lambda ={\left(\mathrm{diag}\left(G+\alpha^{2}I\right)\right)}^{-1}\mathrm{diag}\left(G\right)$$

where $G\overset{\mathrm{def}}{=}U^{T}\mu \mu^{T}U+U^{T}\Sigma U$.

### 4.2. Geometric Duality Implicit Prior Learning

In practice, f(p) is unknown in Equation (14), so we cannot estimate the *a posteriori* probability of Λ. Instead, we could approximate the distribution of f(p) from the geometric duality prior between the LR and HR image patches, rather than from a set of training example images, through the *Gaussian process*.

The obvious advantage of the *Gaussian process* over regression problems is that we can obtain the predictive distribution of the test output sample, such as the mean and covariance, rather than giving a hard assignment. We can directly estimate the output by learning a predictive function g(x):X→Y from training data. Note that, here, x and y are defined at the local patch level, where x are the patches from the HR image and y are the corresponding patches from the LR interpolated image. We use a non-parametric model, which assumes a *Gaussian process* prior $y=g\left(x\right)∼\mathrm{GP}\left(m\left(x\right),k\left(x_{i},x_{j}\right)\right)$ with m(x)=0. The joint distribution of the training outputs and the test outputs is:

(15) $$p\left(y|x\right)∼N\left(0,\left[\begin{array}{c} K(x^{\mathrm{tr}},x^{\mathrm{tr}})\\ K(x^{\mathrm{te}},x^{\mathrm{tr}}) \end{array}\;\begin{array}{c} K(x^{\mathrm{tr}},x^{\mathrm{te}})\\ K(x^{\mathrm{te}},x^{\mathrm{te}}) \end{array}\right]\right)$$

where $y=[y^{\mathrm{tr}},y^{\mathrm{te}}]$, $y^{\mathrm{tr}}=\left[y_{1}^{\mathrm{train}},\cdots ,y_{N}^{\mathrm{train}}\right]$ are *N* training output samples and $y^{\mathrm{te}}=\left[y_{1}^{\mathrm{test}},\cdots ,y_{M}^{\mathrm{test}}\right]$ are *M* test output samples. $x=[x^{\mathrm{tr}},x^{\mathrm{te}}]$ are the corresponding input samples. $K(x^{\mathrm{tr}},x^{\mathrm{te}})$ denotes the N×M matrix of the covariances evaluated at all pairs of training input and test input points and similarly for the other entries $K(x^{\mathrm{tr}},x^{\mathrm{tr}})$, $K(x^{\mathrm{te}},x^{\mathrm{te}})$ and $K(x^{\mathrm{te}},x^{\mathrm{tr}})$. For an input sample $x^{\mathrm{test}}$, the posterior over the output sample $y^{\mathrm{test}}$ has a simple Gaussian form: $p\left(y^{\mathrm{test}}|x^{\mathrm{tr}},y^{\mathrm{tr}},x^{\mathrm{test}}\right)∼N\left(\mu_{y},\Sigma_{y}\right)$, where:

(16) $$\begin{array}{c} \mu_{y}=K\left(x^{\mathrm{te}},x^{\mathrm{tr}}\right){\left(K\left(x^{\mathrm{tr}},x^{\mathrm{tr}}\right)\right)}^{-1}y^{\mathrm{tr}}\\ \Sigma_{y}=K\left(x^{\mathrm{te}},x^{\mathrm{te}}\right)-K\left(x^{\mathrm{te}},x^{\mathrm{tr}}\right){\left(K\left(x^{\mathrm{tr}},x^{\mathrm{tr}}\right)\right)}^{-1}K\left(x^{\mathrm{tr}},x^{\mathrm{te}}\right) \end{array}$$

**Figure 1:**
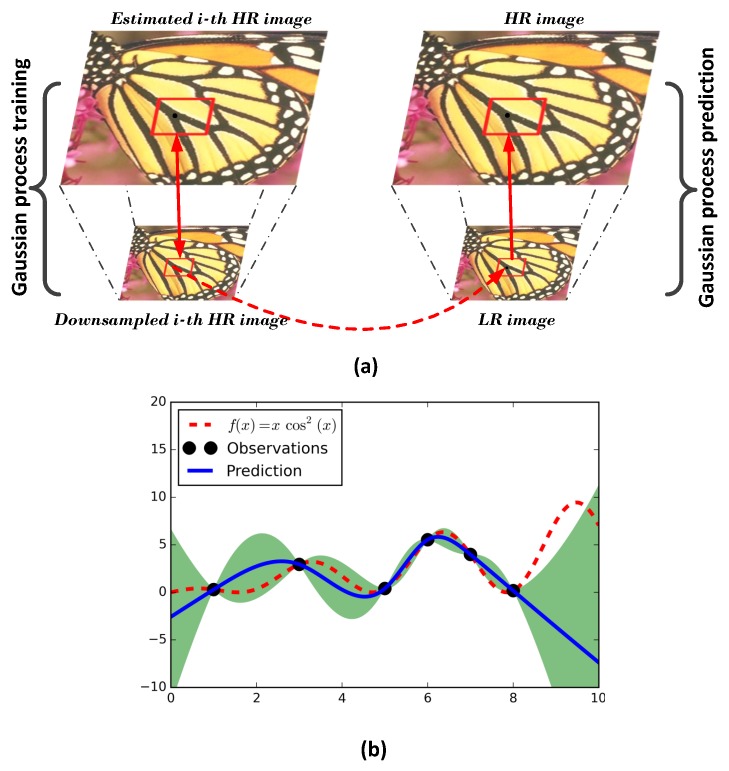
Illustration of the *Gaussian process* for image patch prior learning. (**a**) The overview of image patch prior learning. The left half is the training process, and the right half is the prediction process; (**b**) Example of one-dimensional data. The black points correspond to the observed training data points. The red dotted curve represents the true function. The blue solid curve is the prediction mean, and the green area is the variance.

Figure 1 shows an illustration of the *Gaussian process* for image patches’ prior learning. In our setting, we use *t*-th estimated HR image ${\hat{x}}^{t}$ in the *M-step* as a training output, the corresponding interpolated image (obtained by interpolating the image ${\hat{y}}^{t}$ using the bicubic function, where ${\hat{y}}^{t}=S{\hat{x}}^{t}$ as the training input and the LR interpolated image (obtained by interpolating the LR image y) as the testing input. Each 7×7 patch from the training input and the corresponding patch from the training output form a predictor-target training pair. In order to be more specific according to the local prior, the training is carried out in a 30×30 overlapped region separately from which the training pairs come.

### 4.3. Improving Similar Patches Match by Introducing Rotation Invariance

As mentioned in SubSection 4.1, the dictionary U is computed from the reference patches. Their group matrix P is composed of non-local patches similar to the patch q, where the patch selection/matching is performed by measuring the Euclidean distance similarity between the patch q and each of the patches from its image. The non-local similarity matching is based on the fractal nature of images, which suggests that patches of a natural image recur within the same image.

However, one problem is that the pattern may rarely repeat in the image. Thus, the non-local characteristics will not be sufficiently expressive to cover all of the patches. One expands the internal patch search space by allowing geometric variations achieved by affine transformations [30]. We propose a rotated non-local self-exemplars strategy for similar image patches’ matching and improve the performance.

**Figure 2:**
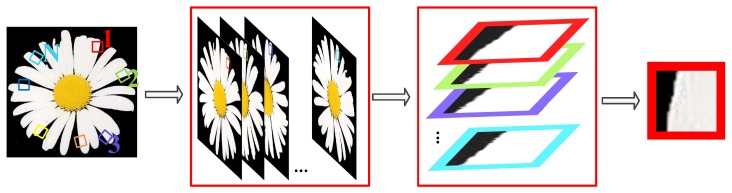
The rotated non-local self-exemplars strategy for the image filtering method.

**Figure 3:**
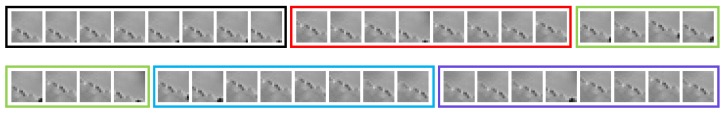
The rotated non-local self-exemplars strategy for the image patches’ matching method. The patches in black box are from non-rotated image (rotation angle θ=0°). The patches in the red box are from the image with rotated angle θ=45°. The patches in the green box are from the image with rotated angle θ=90°. The patches in the blue box are from the image with rotated angle θ=135°. The patches in the purple box are from the image with rotated angle θ=180°.

Our rotated non-local self-exemplars strategy finds that the most similar *k* images patches come from the rotated poses, rather than the original pose in the image. It alleviates the drawback induced by Euclidean distance similarity matching, which is computed by summating the square-error between the targeted pixels and corresponding pixels. By rotating the image, the similar image patches can be successfully matched by the Euclidean distance. In the illustration in Figure 2, we can see that the leftmost is the input noisy image; the rotated images are second from left; the image patches’ matching is accomplished in the third from the left; and the noisy image patch is showed in the rightmost. An example of the rotated non-local self-exemplars strategy is given in Figure 3.

## 5. Experiment Results and Discussion

To demonstrate the superior performance of our proposed super-resolution method, we first compare it to bicubic interpolation and five representative methods in the image super-resolution field on noiseless images. These are Yang’s method based on the sparse representation prior [11], *Gaussian process* regression for super-resolution (GPR) [20], Zhang’s method based on Markov random field prior learning [9], Dong’s method based on adaptive sparse domain selection and adaptive regularization (ASDS) [8] and adjusted anchored neighbor regression (ANR) [31]. Then, a comparison is done on the synthesized noisy images to show the robustness of our algorithm to noise.

### 5.1. Experimental Configuration

In order to evaluate the image super-resolution results with objective measures, the LR images (training or test images) are generated from the original HR image by a 7×7 Gaussian blurring operator with a standard deviation of 1.6 and then downsampled by a factor of three, which is similar to the actual VSN cameras’ imaging. Considering that the HR images of the VSN are hard to acquire, we use the basic open images in experiments. As most VSN applications use grey scale images/videos, we apply our algorithm to the illuminance channel only. For the other two color layers (Cb, Cr), we enlarge them using bicubic interpolation. For the noisy LR images in Section 5.3, the Gaussian noise is added to the LR images generated. As the parameter *α* in the EM algorithm impacts the effectiveness, we empirically set α=0.8δ+1. In the *M-step* HR image estimation procedure, the size of the image patch is set to 7×7, with one overlap pixel between adjacent patches. In the similar patches matching procedure, the number *k* of similar image patches is set to 40. The rotated angle of the non-local self-exemplars strategy is set to θ=0°,45°,90°,135°,180°, respectively. We evaluate the results of various methods both visually and qualitatively in *peak signal to noise ratio* (PSNR) and *structural similarity index measurement* (SSIM). Note that since we only work on the illuminance channel, the reported PSNR and SSIM are carried out only on the illuminance channel. Additionally, we evaluate the super-resolution capability of the different algorithms using twenty benchmark test images used in [31].

### 5.2. Comparison with Six Super-Resolution Algorithms

**Figure 4:**
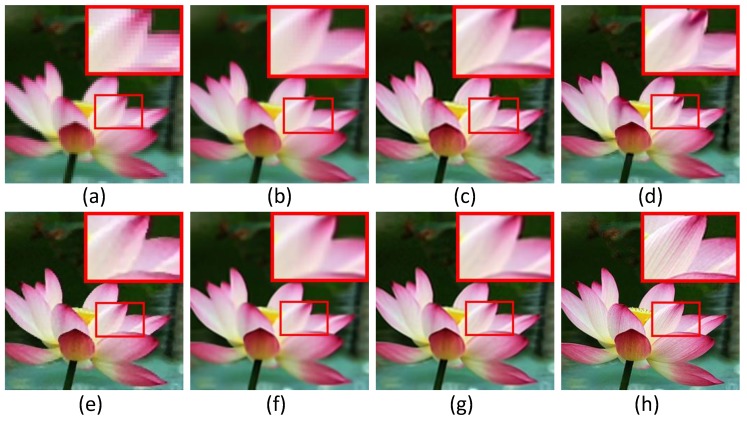
Comparisons with various image super-resolution methods on the image of *Water lily*. (**a**) Bicubic interpolation; (**b**) Yang’s method [11]; (**c**) anchored neighbor regression (ANR) [31]; (**d**) *Gaussian process* regression (GPR) [20]; (**e**) Zhang’s method [9]; (**f**) adaptive sparse domain selection (ASDS) [8]; (**g**) joint-prior image super-resolution (JPISR) method; (**h**) ground-truth.

**Figure 5:**
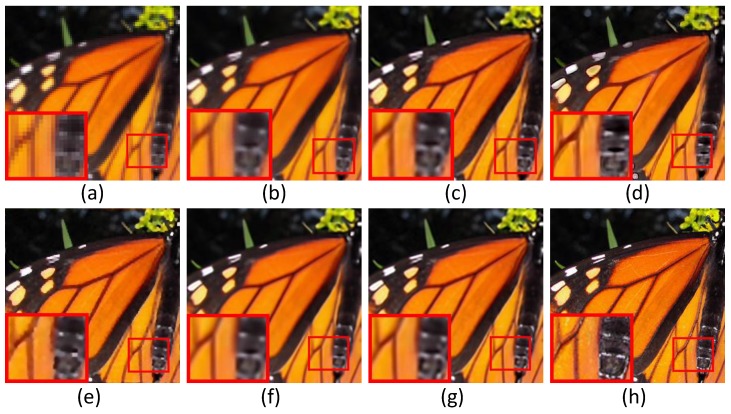
Comparisons with various image super-resolution methods on the image of *Butterfly1*. (**a**) Bicubic interpolation; (**b**) Yang’s method [11]; (**c**) ANR [31]; (**d**) GPR [20]; (**e**) Zhang’s method [9]; (**f**) ASDS [8]; (**g**) JPISR method; (**h**) ground-truth.

**Figure 6:**
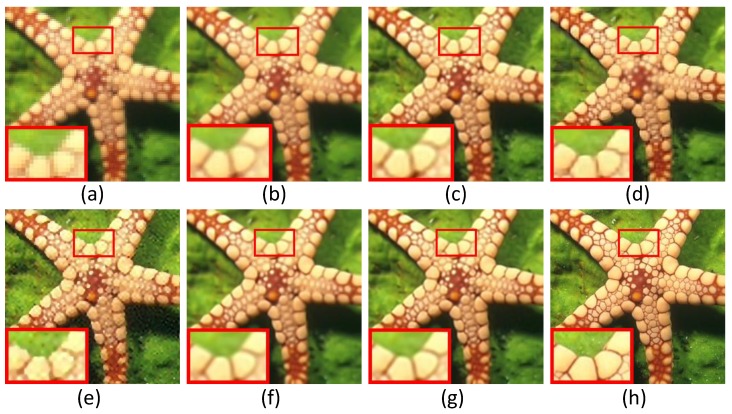
Comparisons with various image super-resolution methods on the image of *Starfish*. (**a**) Bicubic interpolation; (**b**) Yang’s method [11]; (**c**) ANR [31]; (**d**) GPR [20]; (**e**) Zhang’s method [9]; (**f**) ASDS [8]; (**g**) JPISR method; (**h**) ground-truth.

**Figure 7:**
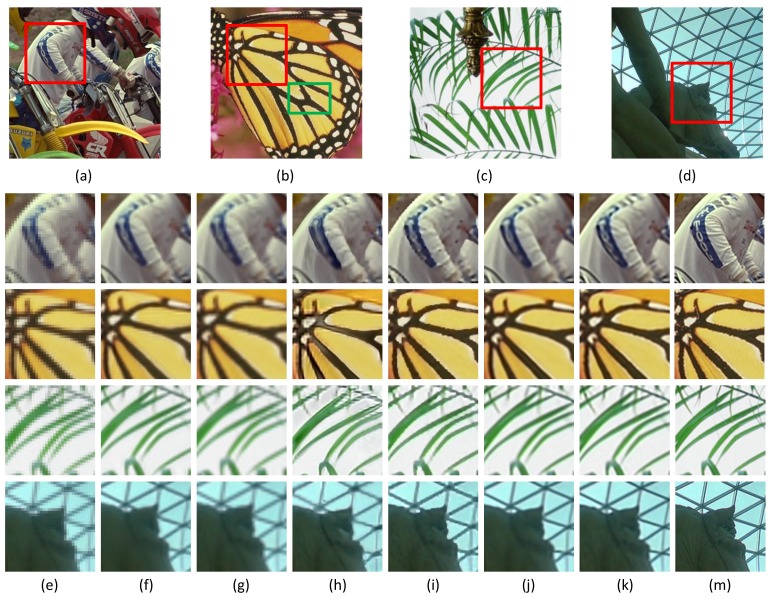
The magnified super-resolution views of the region (red box), where each row represents the same LR image region and each column represents the same method. (**a**) *Bike*; (**b**) *Butterfly2*; (**c**) *Leaves*; (**d**) *Roof*; (**e**) bicubic interpolation; (**f**) Yang’s method [11]; (**g**) ANR [31]; (**h**) GPR [20]; (**i**) Zhang’s method [9]; (**j**) ASDS [8]; (**k**) JPISR method; (**m**) ground-truth.

**Figure 8:**
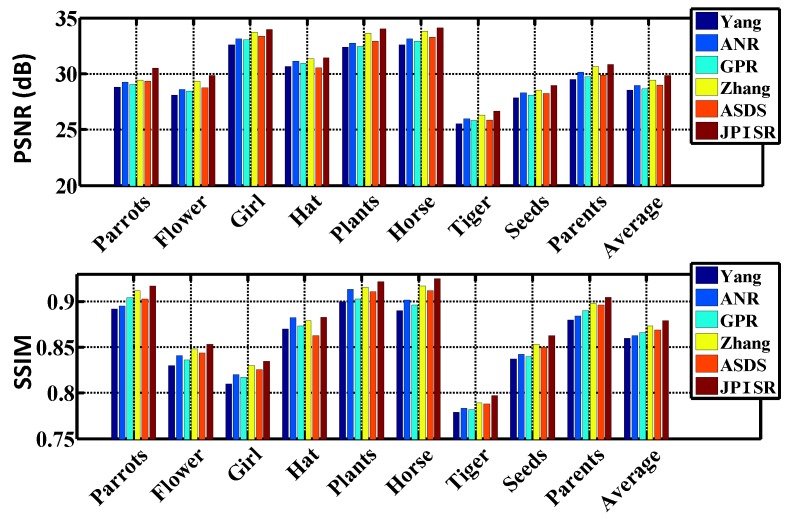
PSNR and SSIM of Yang’s method [11], ANR [31], GPR [20], Zhang’s method [9], ASDS [8] and the JPISR method.

In this subsection, we evaluate the performance of six super-resolution methods (including the bicubic interpolation method) in comparison with the proposed algorithms on twenty benchmark test images used in [31], *i.e.*, *Water lily* (256×256), *Butterfly1* (256×256), *Starfish* (256×256), *Bike* (256×256), *Butterfly2* (256×256), *Leaves* (256×256) and *Roof* (256×256). To visualize the performance difference, we magnify the region (red box) in each test image. Figure 4, Figure 5 and Figure 6 show the visual comparisons. Then, we give the partially enlarged visual views in Figure 7. Figure 8 gives the numerical results. Figure 4a shows the result of the bicubic interpolation algorithm, which includes both visually displeasing blurred textural details and serious jagged artifacts along the edges. Figure 4b shows the result obtained by Yang’s method. Although this method produces sharper edges than bicubic interpolation, there is no further detail added. Figure 4c generates a relative high-quality HR image with many fine details. There are still some unpleasing artifacts along major edges and an over-smoothed region. Figure 4d illustrates the result of the GPR method. This method can produce an HR image of high quality and with rich artifacts. However, the result has both jagged edge artifacts and the preservation of annoying textural details, especially around the edge of the lotus leaf. Figure 4e shows the result obtained by Zhang’s method. It produces over-smoothed results and eliminates much of the image details. It fails to reconstruct fine image edges. Figure 4f illustrates the result of the ASDS method. It performs well in synthesizing many fine details, but there are some noticeable blurring details along dominant edges. Our proposed method (Figure 4g) can produce a visually comparable result to the ASDS method. The enhanced result is more faithful to the original HR image in terms of finer details and sharper edges by exploiting repetitive patterns to suppress the unexpected artifacts that most example learning-based approaches will produce. Figure 5, Figure 6 and Figure 7 validate the above description. To sum up, our method reduces the annoying artifacts and leads to a more faithful super-resolution reconstruction. To further validate our proposed super-resolution algorithm, we present the PSNR and SSIM comparisons of the methods in Figure 8. In the comparative super-resolution methods, Zhang’s method has higher values for PSNR and SSIM. However, its visual effect is the worst. We can see that our proposed method is consistently better than the compared methods, not only for the pleasing visual results, but also the better PSNR and SSIM.

### 5.3. Comparison on Noisy Images

**Figure 9:**
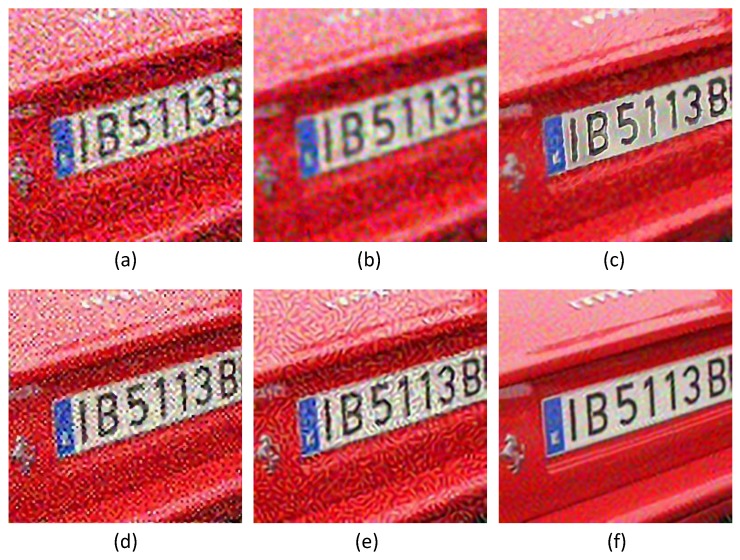
The super-resolution result on LR image *Car tail* with a noise deviation of 20. (**a**) Yang’s method [11]; (**b**) ANR [31]; (**c**) GPR [20]; (**d**) Zhang’s method [9]; (**e**) ASDS [8]; (**f**) JPISR method.

**Figure 10:**
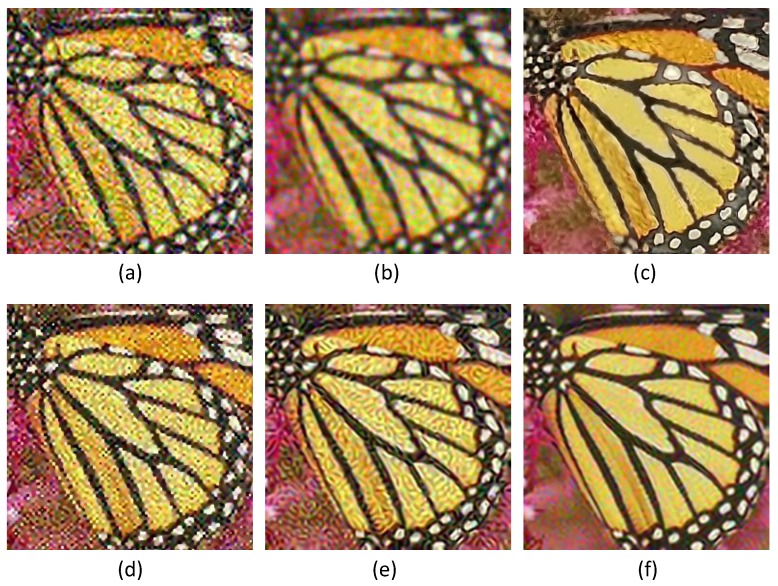
The super-resolution result on LR image *Butterfly 2* with a noise deviation of 20. (**a**) Yang’s method [11]; (**b**) ANR [31]; (**c**) GPR [20]; (**d**) Zhang’s method [9]; (**e**) ASDS [8]; (**f**) JPISR method.

**Figure 11:**
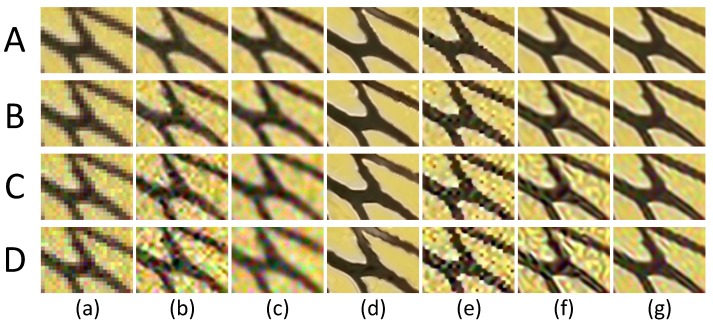
The magnified super-resolution views of region (green box) in Figure 7, where each row represents the same noise variance and each column represents the same method. Rows A, B, C, D are the results with noise deviations of 5, 10, 15, 20, respectively. (**a**) Bicubic interpolation; (**b)** Yang’s method [11]; (**c**) ANR [31]; (**d**) GPR [20]; (**e**) Zhang’s method [9]; (**f**) ASDS [8]; (**g**) JPISR method.

**Figure 12:**
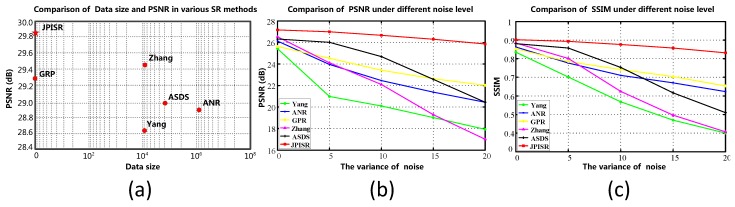
(**a**) Comparison with five super-resolution methods on PSNR values against data sizes; (**b**) comparison of PSNR under different noise levels; (**c**) comparison of SSIM under different noise levels.

In practice, the LR image is often noise corrupted in the VSN due to the working condition, which makes the super-resolution more challenging. We added Gaussian white noise (with standard deviations of 5, 10, 15 and 20, respectively) to the LR images, and the reconstructed HR images are shown in Figure 9, Figure 10 and Figure 11. From Figure 9 and Figure 10, we can see that Yang’s method, ANR, Zhang’s method and the ASDS method are sensitive to noise. The severe noise-caused artifacts can be found around the edges. The GPR method results in over-smoothed HR images. In contrast, the proposed method is more robust to noise. Figure 11 gives the more detailed visual results for comparison. Meanwhile, we give the numerical results under different noise levels in Figure 12b,c. It clearly shows that JPISR has a distant capability of suppressing noise, especially for sever noise.

### 5.4. Comparison of Data Size with PSNR

It is well known that most super-resolution methods require large external datasets for training. However, this is not practical in the VSN. in contrast, JPISR uses the non-local group-sparsity and the geometric duality for joint-prior learning, which does not rely on the external training data. Figure 12a gives the comparison of data sizes and PSNR values of five methods in the experiment. We can see that all other methods need enormous training data, except for JPISR and GPR. However, our method still achieves the highest PSNR value, even compared to Zhang’s method.

## 6. Conclusions

Due to its flexibility and low cost, the VSN has attracted more interest in the last few years and is expected to play a major role in the evolution of smart sensing, data collaborative processing and communication capabilities. Unfortunately, in many cases, the images captured by live cameras are often of low resolution with noise due to the environment or equipment limitations. To make the quality of the captured image more suitable for analysis in various surveillance applications, we proposed a novel framework of prior-adaptive image super-resolution based on the EM algorithm. It inherently combined the super-resolution characteristics (implicit prior) with the image filtering method (explicit prior) to upscale and denoise the LR images captured from the low-cost visual nodes in the VSN. In addition, the proposed joint-prior learning does not rely on the external training data, which is versatile for hostile environments, such as video surveillance in the wild, traffic monitoring, *etc.*

Although our method shows potential for VSN image SR, two aspects need to be considered in our future research. First, our proposed method is relatively time consuming, which cannot meet the real-time requirement. Second, all SR methods can only be trained under a specific degrading process, which restricts practical use. In future work, we will do a parallel implementation on GPU acceleration. Meanwhile, we plan to estimate the camera’s degradation parameters by the blind restoration strategy.
